# Differences between hospitals in attainment of parathyroid hormone treatment targets in chronic kidney disease do not reflect differences in quality of care

**DOI:** 10.1186/1471-2369-13-82

**Published:** 2012-08-06

**Authors:** Mieke J Peeters, Arjan D van Zuilen, Jan AJG van den Brand, Peter J Blankestijn, Marc AGJ ten Dam, Jack FM Wetzels

**Affiliations:** 1 Department of Nephrology, Radboud University Nijmegen Medical Centre, Nijmegen, The Netherlands; 2 Department of Internal Medicine, Canisius Wilhelmina Hospital, Nijmegen, The Netherlands; 3 Department of Nephrology, University Medical Center Utrecht, Utrecht, The Netherlands

**Keywords:** Chronic kidney disease, Parathyroid hormone, Quality of care, Treatment targets

## Abstract

**Background:**

Transparency in quality of care (QoC) is stimulated and hospitals are compared and judged on the basis of indicators of performance on specific treatment targets. In patients with chronic kidney disease, QoC differed significantly between hospitals. In this analysis we explored additional parameters to explain differences between centers in attainment of parathyroid hormone (PTH) treatment targets.

**Methods:**

Using MASTERPLAN baseline data, we selected one of the worst (center A) and one of the best (center B) performing hospitals. Differences between the two centers were analyzed from the year prior to start of the MASTERPLAN study until the baseline evaluation. Determinants of PTH were assessed.

**Results:**

101 patients from center A (median PTH 9.9 pmol/l, in 67 patients exceeding recommended levels) and 100 patients from center B (median PTH 6.5 pmol/l, in 34 patients exceeding recommended levels), were included. Analysis of clinical practice did not reveal differences in PTH management between the centers. Notably, hyperparathyroidism resulted in a change in therapy in less than 25% of patients. In multivariate analysis kidney transplant status, MDRD-4, and treatment center were independent predictors of PTH. However, when MDRD-6 (which accounts for serum urea and albumin) was used instead of MDRD-4, the center effect was reduced. Moreover, after calibration of the serum creatinine assays treatment center no longer influenced PTH.

**Conclusions:**

We show that differences in PTH control between centers are not explained by differences in treatment, but depend on incomparable patient populations and laboratory techniques. Therefore, results of hospital performance comparisons should be interpreted with great caution.

## Background

Patients with chronic kidney disease (CKD) are at increased risk of developing cardiovascular disease [1-3]. Multiple traditional and non-traditional risk factors contribute to this increased cardiovascular risk. In recent years an important role of disturbances in bone and mineral metabolism has been established [4-8]. Patients with CKD are characterized by variable degrees of vitamin D deficiency, hyperparathyroidism, hypercalcemia and hyperphosphatemia. These abnormalities contribute to soft tissue and vascular calcification, and ensuing cardiovascular injury [9]. Parathyroid hormone (PTH) level is already elevated in patients with mild CKD and prevalence of hyperparathyroidism rises substantially when glomerular filtration rate (GFR) decreases [10,11]. Consequently, PTH is one of the most deviant laboratory parameters in CKD patients.

The 2003 K/DOQI clinical practice guideline addressed the treatment of CKD-bone and mineral disorder and defined treatment targets for calcium, phosphate, and PTH [12]. Newer guidelines with different targets were recently published [13]. Defining treatment goals is only one aspect of quality of care (QoC). Health care authorities, health insurance companies, as well as organizations of health care professionals have introduced benchmarking as a way to compare and improve QoC [14-16]. Treatment centers are asked to provide figures on treatment targets, complication rates, and survival rates. Hospital performance is determined on the basis of these figures.

Nevertheless, implementation of guidelines is rather difficult and treatment goals are often not met [17-19]. We recently showed that in CKD patients QoC, defined as attainment of treatment targets, differed significantly between treatment centers [20,21]. Differences were not explained by available patient characteristics. We hypothesized that detailed comparison of all aspects of treatment in these centers may help to define parameters that are associated with QoC, and eventually improve performance. In the present study, we compared two centers that differed in the attainment of the PTH treatment goal in CKD patients and explored possible explanations.

## Methods

The MASTERPLAN (Multifactorial Approach and Superior Treatment Efficacy in Renal Patients with the Aid of Nurse practitioners) study [Trial registration ISRCTN registry: 73187232] is a randomized controlled trial conducted in nine centers with a nephrology department in The Netherlands, evaluating the added value of nurse practitioner care in reducing cardiovascular events and attenuating kidney function decline in patients with prevalent CKD. Rationale and design have been published elsewhere [22,23]. Ethics committee approval was obtained as well as written informed consent of all participants. Between April 2004 and December 2005, patients were enrolled.

We recently analyzed baseline data and evaluated QoC, defined as achievement of treatment goals. We noted significant differences between treatment centers for various treatment goals such as blood pressure, cholesterol, and PTH [20]. In the present study we focused on management of PTH and selected one of the worst (center A) and one of the best (center B) performing hospitals. Study design can be described as a nested case control study (on hospital level).

PTH is one of the quality indicators of the Dutch Federation of Nephrology. It is, therefore, a usual quality of care target.

PTH was defined as exceeding recommended levels if > 7.7 pmol/l in patients with estimated glomerular filtration rate (eGFR) ≥ 30 ml/min/1.73 m^2^, if > 12.1 pmol/l in patients with eGFR 15–29 ml/min/1.73 m^2^, and if > 33.0 pmol/l in patients with eGFR < 15 ml/min/1.73 m^2^, based upon the then available CKD-bone and mineral disorder guidelines [12].

We used the baseline clinical and laboratory data of the patients as available according to the MASTERPLAN study protocol [23]. We retrieved additional data from the medical records, using a form, specifically addressing known determinants of PTH [24,25] and characteristics of treatment in the year before the baseline MASTERPLAN visit (number of patient visits, number of laboratory tests performed, adjustment of treatment affecting PTH levels). The additional data collection for the present study, was possible on the basis of the before mentioned ethics committee approval and informed consent.

Initially eGFR was calculated using the abbreviated MDRD formula [26] (MDRD-4). In addition the six-variable MDRD formula [27] (MDRD-6) and the CKD-EPI equation [28] were used in order to achieve a more accurate estimation of the GFR. We also compared the serum creatinine assays between the hospitals. The hospitals used different Jaffé methods to measure serum creatinine. Both methods were compared to one enzymatic method (Roche Diagnostics). The following equations were developed. For center A: enzymatic creatinine = 1.266 x Jaffé creatinine - 29. For center B: enzymatic creatinine = (Jaffé creatinine - 28) / 0.93.

In center A PTH was measured by an immunoluminometric assay (Nichols Institute, San Clemente, USA). In center B an immunoradiometric assay from the same manufacturer was used. Since both methods use antibodies directed against the same regions of the PTH molecule and reference values are similar we consider the methods comparable. This is confirmed by Souberbielle *et al.*, as they found no significant difference in measured PTH concentration between both methods [29].

Proteinuria and smoking data were missing in two patients, income data were missing in 34 patients. Two analyses were performed: one without cases with missing values and another in which missing data were imputed by single imputation. In proteinuria and income median values were imputed, in smoking the mode was used. The presented data are after imputation.

In the analysis of characteristics of treatment in the year before MASTERPLAN baseline (management of hyperparathyroidism), we excluded patients who were under specialist physician care for less than six months prior to study enrollment. We used the six month criterion since we aimed to evaluate differences in QoC between hospitals and not between general practitioners.

Characteristics are given for the study population by treatment center and expressed as means (standard deviation) or proportions. Medians [interquartile range] are presented for variables with a skewed distribution. Differences between the two treatment centers were studied using an independent-samples T test for continuous variables and a chi-square test for categorical variables. The natural logarithm (Ln) of PTH had a normal distribution and was used for correlation analyses. Correlation with several potential determinants was studied using a Pearson correlation coefficient for continuous variables and a Spearman’s rho correlation coefficient for categorical variables. If necessary, the Ln of continuous variables was used to obtain a normal distribution. Multivariate analyses using stepwise backward linear regression models were performed to find determinants of PTH. Variables with univariate associations and potential confounders were included. Criteria for exclusion and re-inclusion were p ≥ 0.10 and p < 0.05 respectively. Variables were excluded from the final multivariate model if they did not contribute considerably to the explained variance (change in R^2^ < 5%). Multivariate Poisson regression models were used to construct prediction models for meeting the PTH treatment target. Variables with univariate associations were included. All p-values were two-sided, and for all tests p-values less than 0.05 were considered to indicate statistical significance. The univariate analyses were performed with SPSS 16.0 (SPSS Inc., Chicago, USA). The multivariate analyses were performed with Stata 10.1 (StataCorp LP, College Station, USA).

## Results

Medical records of 101 patients from center A and 100 patients from center B were studied. Center A is a university clinic with 953 beds and center B is a non-university clinic with 653 beds. Both centers are teaching hospitals that offer a full range of nephrology treatment including kidney replacement therapy and are involved in the care of kidney transplant recipients. The two centers are located in the same region of The Netherlands.

Characteristics of the study population are given in Table 1. The majority of patients were male (71%) and Caucasian (96%). Twenty one percent of the patients were kidney transplant recipients. Median plasma PTH level was 8.7 pmol/l. By definition the median PTH level and the number of patients with PTH exceeding recommended levels were significantly different between the two treatment centers (Table 1).

**Table 1:** Characteristics of study population

	**Center A (n = 101)**	**Center B (n = 100)**	**p**
Plasma PTH (pmol/l)	9.9 [6.7-15.5]	6.5 [3.6-11.0]	<0.001
PTH exceeding recommended levels	67 (66%)	34 (34%)	<0.001
Age (years)	52.2 (12.7)	58.8 (11.8)	<0.001
Male sex	68 (67%)	75 (75%)	0.23
Caucasian race	99 (98%)	93 (93%)	0.09
Cause of kidney disease			<0.001
Glomerulonephritis	34 (34%)	17 (17%)	
Diabetic nephropathy	9 (9%)	4 (4%)	
Renovascular	4 (4%)	15 (15%)	
Interstitial nephritis	25 (25%)	6 (6%)	
Congenital (including PKD)	20 (20%)	5 (5%)	
Different/unknown	9 (9%)	53 (53%)	
Kidney transplant recipient	33 (33%)	9 (9%)	<0.001
History of diabetes mellitus	20 (20%)	13 (13%)	0.19
Income (euros/month)	2108 (489)	2030 (482)	0.26
BMI (kg/m^2^)	27.0 (5.0)	28.6 (4.5)	0.01
Smoking	15 (15%)	19 (19%)	0.43
Serum calcium (mmol/l)	2.40 (0.13)	2.40 (0.12)	0.71
Serum phosphate (mmol/l)	1.06 (0.25)	1.01 (0.22)	0.15
Serum urea (mmol/l)	13.5 [10.1-17.6]	9.8 [7.2-15.2]	<0.001
Serum albumin (g/l)	39 (4)	42 (3)	<0.001
Proteinuria (g/24 h)	0.6 [0.1-1.3]	0.3 [0.2-0.9]	0.10
Serum creatinine (μmol/l)	168 (53)	184 (60)	0.05
eGFR (MDRD-4, ml/min/1.73 m^2^)	40.3 (12.4)	36.3 (11.1)	0.02
Alphacalcidol use	16 (16%)	13 (13%)	0.57
Calciumcarbonate use	13 (13%)	5 (5%)	0.05
Alphacalcidol and/or calciumcarbonate use	21 (21%)	16 (16%)	0.38
Furosemide use	17 (17%)	9 (9%)	0.10
Thiazide use	22 (22%)	46 (46%)	<0.001
Number of drugs	5.82 (3.90)	5.73 (2.85)	0.85
Season of blood draw			0.34
Winter	21 (21%)	15 (15%)	
Spring	24 (24%)	17 (17%)	
Summer	24 (24%)	30 (30%)	
Fall	32 (32%)	38 (38%)	

Values are given as mean (SD), n (%), or median [interquartile range].

PTH: parathyroid hormone; PKD: polycystic kidney disease; BMI: body mass index; eGFR: estimated glomerular filtration rate; MDRD: modification of diet in renal disease.

### Management of hyperparathyroidism

Table 2 shows the treatment characteristics of patients in the year before entry in the MASTERPLAN study. Three patients from center A were excluded from this analysis and 18 patients from center B, because they were under specialist physician care for less than six months at MASTERPLAN baseline. In center A patients visited their physicians more often, and more laboratory tests were performed, although the number of PTH tests was not significantly different. In center A more different nephrologists and general internists were involved in the patient’s treatment. PTH levels were not measured in 29-39% of patients in the six-twelve months before the start of MASTERPLAN. Of the patients with known PTH levels 29 patients in center A and 27 patients in center B had a PTH level exceeding recommended levels. There was no significant difference in the way that center A and center B handled the patients with PTH exceeding recommended levels (Table 2). Specifically, hyperparathyroidism resulted in a change in therapy in less than 25% of patients.

**Table 2:** Characteristics of treatment in the year before MASTERPLAN baseline in center A and B

	**Center A (n = 98)**	**Center B (n = 82)**	**p**
No. of patient visits	6.73 (6.21)	3.11 (1.41)	<0.001
No. of different nephrologists/ internists	1.92 (1.63)	1.10 (0.30)	<0.001
No. of patient letters written	0.70 (0.48)	0.65 (0.51)	0.43
No. of laboratory tests			
Serum calcium	4.05 (4.31)	2.39 (1.77)	0.001
Serum phosphate	3.97 (4.14)	2.30 (1.68)	0.001
Plasma PTH	0.92 (1.48)	1.01 (0.88)	0.61
Serum creatinine	8.59 (9.80)	5.32 (4.24)	0.01
PTH level known	60 (61%)	58 (71%)	0.18
PTH exceeding recommended levels	29 (30%) (n = 60)	27 (47%) (n = 58)	0.85
If PTH was exceeding recommended levels	(n = 29)	(n = 27)	
Already on medication	8 (28%)	6 (22%)	0.64
Adjustment of treatment	4 (14%)	2 (7%)	0.44
Start treatment	3 (10%)	4 (15%)	0.61
PTH still exceeding recommended levels at MASTERPLAN baseline	26 (90%)	21 (78%)	0.23
Change in serum creatinine (μmol/l)	1.1 (31.1)	8.2 (23.6)	0.09
Hospitalization (at least once)	22 (22%)	12 (15%)	0.18
Surgery (at least once)	13 (13%)	5 (6%)	0.11

Only patients who were under the care of a specialist physician (nephrologist or internist) for at least 6 months before the MASTERPLAN study were analyzed.

Values given are mean (SD) or n (%).

PTH: parathyroid hormone.

### Determinants of PTH

Correlation analyses showed that cause of kidney disease (p = 0.03), kidney transplant status (p < 0.001), income (p = 0.02), serum calcium (p = 0.03), serum phosphate (p < 0.001), eGFR (p < 0.001), treatment center (p < 0.001), and furosemide use (p = 0.01) were potential determinants of PTH (Table 3). As shown in Table 1, distribution of cause of kidney disease, kidney transplant status, and eGFR by MDRD-4 differed significantly between the two centers.

**Table 3:** Correlation between plasma (Ln)PTH levels and potential determinants of plasma PTH

	***r***	***r***_***s***_	**p**
Age (years)	-.10		0.16
Male sex		-.01	0.89
Caucasian race		-.07	0.34
Cause of kidney disease renovascular or different/unknown^*^		-.15	0.03
Kidney transplant		.27	<0.001
Diabetes mellitus		.02	0.82
Income (euros/month)	-.16		0.02
BMI (kg/m^2^)	.00		0.99
Smoking		.01	0.94
Serum calcium (mmol/l)	-.16		0.03
Serum phosphate (mmol/l)	.29		<0.001
(Ln)Proteinuria (g/24u)	.12		0.08
Serum creatinine (μmol/l)	.48		<0.001
eGFR (MDRD-4, ml/min/1.73 m^2^)	-.48		<0.001
eGFR (MDRD-6, ml/min/1.73 m^2^)	-.55		<0.001
Center A		.27	<0.001
Alphacalcidol use		.18	0.01
Calciumcarbonate use		.17	0.02
Alphacalcidol and/or calciumcarbonate use		.21	0.003
Furosemide use		.19	0.01
Thiazide use		-.01	0.87
Number of drugs	0.35		<0.001
Blood drawn in summer or fall^*^		-.08	0.25

Ln: natural logarithm; PTH: parathyroid hormone; *r*: Pearson correlation coefficient; *r*_*s*_: Spearman’s rho correlation coefficient; BMI: body mass index; eGFR: estimated glomerular filtration rate; MDRD: modification of diet in renal disease.

^*^Patients classified as having a renovascular or different/unknown cause of kidney disease on average had the lowest (Ln)PTH levels. Therefore these categories were combined. The same holds for patients with blood drawn in summer or fall.

The factors that were univariately associated with plasma PTH were all included in multivariate analyses. Potential confounders that were also included were age, sex, race, history of DM, BMI, smoking status, proteinuria, thiazide use, and season of blood draw. Although alphacalcidol and/or calciumcarbonate use and number of drugs were positively correlated with PTH, they were not included in multivariate models because of reverse causation (patients who have higher PTH levels more often use alphacalcidol, however the drug does not lead to higher PTH levels).

Table 4 shows the results of multivariate linear regression analysis by which determinants of PTH were identified. Kidney transplant status, MDRD-4, and treatment center were independent predictors of plasma PTH level.

**Table 4:** Multivariate linear regression (backward) analyses on predictors of plasma (Ln)PTH level

**eGFR calculated with**	**Model with or without treatment center**	**Explained variance**	**Independent predictors**	**Linear regression-coefficient [95% CI]**
MDRD-4 before creatinine conversion	Including treatment center	R^2^ = 0.42	Center	0.49 [0.31-0.68]
			Kidney transplant	0.55 [0.33-0.78]
			eGFR (MDRD-4)	−0.04 [−0.05 - -0.03]
	Without treatment center	R^2^ = 0.34	Kidney transplant	0.72 [0.48-0.95]
			eGFR (MDRD-4)	−0.04 [−0.04 - -0.03]
MDRD-6 before creatinine conversion	Including treatment center	R^2^ = 0.45	Center	0.36 [0.19-0.54]
			Kidney transplant	0.54 [0.32-0.76]
			eGFR (MDRD-6)	−0.04 [−0.05 - -0.03]
	Without treatment center	R^2^ = 0.40	Kidney transplant	0.67 [0.46-0.89]
			eGFR (MDRD-6)	−0.04 [−0.05 - -0.03]
MDRD-6 after creatinine conversion	Including treatment center	R^2^ = 0.45	Center	0.13 [−0.05-0.31]
			Kidney transplant	0.54 [0.32-0.76]
			eGFR (MDRD-6)	−0.03 [−0.04 - -0.03]
	Without treatment center	R^2^ = 0.44	Kidney transplant	0.59 [0.38-0.79]
			eGFR (MDRD-6)	−0.03 [−0.04 - -0.03]

The difference in R^2^ between the model with and without treatment center illustrates the contribution of the treatment center to the explained variance. The linear regression coefficient reflects the association between the independent predictor and (Ln)PTH. eGFR is a very important determinant, since for every extra ml filtration per minute, PTH decreases by three percent (based on the final model using MDRD-6 after creatinine conversion).

Ln: natural logarithm; PTH: parathyroid hormone (pmol/l); eGFR: estimated glomerular filtration rate (ml/min/1.73 m^2^); MDRD: modification of diet in renal disease; CI: confidence interval.

### Role of GFR

Glomerular filtration rate is a well known determinant of PTH. Various methods can be used to estimate GFR. Since there were significant differences between center A and B in serum urea and albumin, we considered that MDRD-6 (which includes these variables) might give a better estimation of GFR than MDRD-4 and subsequently predict plasma PTH level more accurately. Estimated GFR by MDRD-6 was 37.7 (SD 12.0) for center A and 37.0 ml/min/1.73 m^2^ (SD 12.2) for center B, p = 0.69. Table 4 shows that, while the explained variance increased, the center effect was reduced when MDRD-6 instead of MDRD-4 was used in the model. We also used the CKD-EPI equation, but this did not improve the model, nor reduced the center effect (data not shown).

Serum creatinine is an important parameter in the MDRD formulas. After conversion of serum creatinine to enzymatic values, mean eGFR by MDRD-6 was 35.7 ml/min/1.73 m^2^ in center A and 42.1 ml/min/1.73 m^2^ in center B (p = 0.002).

We subsequently used MDRD-6 after creatinine conversion in multivariate analysis, and found that treatment center was no longer a significant predictor of PTH (Table 4).

Since attainment of the PTH treatment target not only depends on PTH, but also on eGFR, we extended the multivariate analyses to determine predictors of meeting the PTH treatment target. The results were similar as reported in Table 4 (data not shown).

## Discussion

In our study we compared two hospitals that differed in the attainment of the PTH treatment goal and explored possible explanations. We showed that the apparent differences were explained by incomparable patient populations and laboratory techniques, and the use of the abbreviated MDRD formula to estimate GFR. After correction for kidney transplant status and with the use of the MDRD-6 formula with calibrated creatinine to estimate GFR, treatment center no longer influenced the attainment of the PTH treatment goal.

We evaluated potential differences in treatment which might explain any difference in attainment of the PTH target. We focused on characteristics of the treatment given to patients in the year before the start of the MASTERPLAN study. Plantinga *et al.* have shown that more frequent patient-physician contacts in patients with end-stage renal disease are positively associated with the achievement of clinical performance targets, including targets of bone and mineral disorder [30]. One would therefore expect that patients in center A, the ‘worst performing’ hospital, visited their physician less often and had less laboratory tests done. Table 2 shows that the opposite was true. Admittedly, the number of visits and laboratory tests may be dictated by patient morbidity and disease history and not necessarily reflect QoC, e.g. kidney transplant recipients in general need more frequent control. In addition, there were no differences in the way both centers handled the patients with PTH values exceeding recommended levels.

Table 2 also shows that relatively few PTH tests were performed, while PTH is one of the most deviant laboratory values in CKD patients. In both centers in only a small number of patients with PTH exceeding recommended levels, treatment was adjusted or started. Thus, although guidelines give attention to treatment of CKD- bone and mineral disease, and although treatment targets are well defined, physicians are insufficiently aware of the importance of adequate treatment of hyperparathyroidism: our data point to therapeutic inertia towards the PTH treatment target.

In univariate analysis several factors were potential determinants of PTH. Multivariate analyses showed that many of these factors did not independently predict PTH levels. Renal function on the basis of calibrated creatinine values and kidney transplant status are the most important determinants of attainment of the PTH treatment target.

As mentioned before, GFR is a well known determinant of PTH and increases in PTH occur early in the course of renal insufficiency. We observed an inverse relationship between PTH and eGFR (Table 3). Although eGFR proved to be an important, independent predictor of PTH, in the initial analysis the differences in PTH levels between centers could not be explained by differences in eGFR. However, all formulas for estimating GFR have limitations. We showed that MDRD-4 is invalid in patients with proteinuria, where MDRD-6 proved better [31]. MDRD-6 superiority was also shown in kidney transplant recipients [32]. Since MDRD formulas critically depend on the measurement of serum creatinine, differences between serum creatinine assays affect their performance. Therefore, recent guidelines suggest to use calcibrated serum creatinine values [33].

Our data clearly show that the use of the MDRD-6 formula reduced the center effect. Moreover, when using the MDRD-6 formula and calibrated serum creatinine values, there were no longer significant differences in attainment of PTH treatment targets between the centers.

From these findings we conclude that it is not always valid to use the abbreviated MDRD formula instead of the six-variable MDRD formula, especially in analyses in which GFR plays a central role. Moreover, comparable creatinine assays must always be used. Physicians should be aware of the limitations of formulas for estimating GFR, especially of the MDRD-4 formula, since this formula is extensively used (in The Netherlands and also in many other countries).

Admittedly, although the results may be explained by the better performance of the MDRD-6 formula as measure of real GFR, we cannot exclude that other factors are involved. The MDRD-6 formula incorporates serum albumin concentration. It is known that there is an inverse relationship between plasma calcidiol levels and magnitude of proteinuria, and thus hypoalbuminemia, because of loss of vitamin D metabolites and vitamin D binding protein in the urine in patients with proteinuria [34,35]. Low plasma calcidiol levels are related to higher PTH concentrations [36-39].

It is well known that hyperparathyroidism often persists for many years after kidney transplantation [40,41]. Treatment with vitamin D compounds is complicated, since hyperparathyroidism in post-transplant patients is usually associated with hypercalcemia [40]. Consequently, mean PTH level was higher in kidney transplant recipients and only a smaller number of kidney transplant patients achieved the PTH treatment goal. In center A more kidney transplant recipients were treated than in center B.

Insight and transparency in QoC is becoming more and more important. Various indicators are used to assess hospital performance, for example attainment of treatment targets, as in our study. Benchmarking has become a way to compare and judge treatment centers. Ranking hospitals on the basis of performance indicators is supposed to give health care professionals, insurance companies as well as (associations of) patients insight in QoC. The results of our study question the reliability of these rankings and other hospital performance comparisons.

There are several difficulties associated with hospital performance comparisons: definitions are not always the same [16,42], laboratory assays vary [18], data quality is variable between hospitals and even within one hospital [16,42,43]. Another problem is patient case mix [16,18,42,43]. Patient age, race, severity of illness, and comorbidity all influence the outcome of care. Retrospective risk adjustment can only partly adjust for all these factors. There will always be additional residual confounding [42,43]. Moreover, random variation has to be taken into account [16,42-45]. Therefore, when describing results of hospital performance comparisons, confidence intervals should be provided to give insight into the influence of random variation [16,44]. The practice of summarizing several performance indicators in one composite score adds to the unreliability of performance measures since small differences in methods of constructing the composite score can have substantial impact on the results [44]. Finally, whether performance indicators provide a true reflection of QoC is questionable. Performance indicators represent mainly technical aspects, while the humane side of health care and traditional components of caring, essential when talking about QoC, are ignored [46,47].

A major limitation of our study is that we compared only two hospitals. The extent to which the findings can be generalized beyond the centers and cases studied, is unknown. Another limitation is the lack of plasma calcidiol levels. Since plasma calcidiol and PTH are inversely related [36-39], differences in calcidiol levels can have important consequences for PTH concentrations. Other unknown, and possibly influencing, factors are patient compliance, FGF-23 levels, and dietary intake of calcium, phosphate, protein and vitamin D, including over-the-counter vitamin pills.

## Conclusions

In conclusion, the observed differences in hospital performance on PTH management between center A and center B in patients with CKD are explained by incomparable patient populations and laboratory techniques, and use of the abbreviated MDRD formula to estimate GFR. We propose the use of the MDRD-6 formula to estimate GFR in analyses when variables are critically dependent on glomerular filtration rate. Our study shows that great caution is required in interpreting the results of hospital performance comparisons. Uncritical use of these measures can result in treatment centers being wrongly classified in terms of performance.

## Competing interests

The authors declare that they have no competing interests.

## Authors’ contributions

PB en JW are principal investigators of the MASTERPLAN study, MD is the local investigator. JW, AZ, and MP designed the study. JB and MP analysed the data. Interpretation of data was done by JW, JB, and MP. MP drafted the article, the other authors revised it. All authors read and approved the final manuscript.

## Pre-publication history

The pre-publication history for this paper can be accessed here:

http://www.biomedcentral.com/1471-2369/13/82/prepub
